# Characterization and Control of Hidden Micro-Oxygenation in the Winery: Wine Racking

**DOI:** 10.3390/foods10020386

**Published:** 2021-02-10

**Authors:** Ignacio Nevares, Ainara Fernández-Díaz, Maria del Alamo-Sanza

**Affiliations:** 1 Department of Agricultural and Forestry Engineering, UVaMOX, Universidad de Valladolid, Unidad Asociada al CSIC, 34001 Palencia, Spain; ainara.fernandez@uva.es; 2 Department of Analytical Chemistry, UVaMOX, Universidad de Valladolid, Unidad Asociada al CSIC, 34001 Palencia, Spain

**Keywords:** oxygen, purging, blanketing, free SO_2_, racking, hose, inert gases, connectors

## Abstract

Transferring wine is a common operation in most of the wineries in the world that depends mainly on the equipment and materials used. These contributions are widely unknown, and their knowledge is of vital importance to controlling the winemaking process. This work presents the results of characterizing the oxygen supply due to the use of hoses of different materials (Butyl Rubber, IIR; Nitrile Butadiene Rubber, NBR; Ethylene Propylene Diene Monomer rubber, EPDM; Ultra-high-molecular-weight polyethylene, UHMW; Natural Rubber, NR), dimensions (DN32; DN50), connectors (DIN 11851, Tri-CLAMP) with gaskets of different materials (NBR; EPDM; Fluorocarbon, FKM/FPM; silicone rubber, Q/VMQ; Polytetrafluoroethylene, PTFE). In addition, the use of different inert gases (N_2_, CO_2_, Ar, N_2_ + CO_2_ and Ar + CO_2_) for air purging of hoses and tanks, as well as for ullage blanketing during tank-to-tank racking, and their economic impact were studied. The results indicated that the IIR hoses had the least amount of O_2_ added to the liquid and that the Tri-clamp connectors were generally more airtight, with the FKM seals standing out. The most recommended inert gas was CO_2_ when the type of wine allows it, N_2_ being the most recommended in any case. When all these recommendations were used together the addition of O_2_ during tank-to-tank racking was drastically reduced.

## 1. Introduction

Exposure to oxygen can influence the final quality of wines. Its solubility in musts or wines depends on factors such as temperature, atmospheric pressure, ethanol concentration and the presence of particles in suspension in the wine [1]. Under conditions of 20 °C and at atmospheric pressure, the scarce data in the literature estimate that 8.4 mg/L of dissolved oxygen is necessary to bring wine to saturation [2].

The impact of oxygen on wine is related to aspects such as the modification of phenolic compounds, the modification of the aromatic fraction and the consequent decrease in varietal aromas, the appearance of oxidative notes and effects on the growth and multiplication of microorganisms [3]. When red wine is subjected to controlled oxygen exposure, multiple beneficial results have been obtained in color stabilization, reduction of astringency and bitterness in addition to the use of lower doses of sulfur [4,5].

In the vinification process, from the crushing of the grapes to the bottling of the final wine, there are multiple stages where the concentration of dissolved oxygen is increased, either by treatments carried out or by the use of equipment [6,7,8,9,10], the contribution of oxygen being inevitable. There are processes with a greater risk of causing an increase in dissolved oxygen in the wine, such as racking, filtration, tartaric stabilization by cold and bottling. Under normal conditions, this contribution is consumed mainly by the free SO_2_ (FSO_2_) and by the oxidizable components of the wine itself [11]. In wine racking, uncontrolled oxygen addition ranges from 0.37 to 1.81 mg/L of dissolved oxygen [5,10], depending on many factors. Operating speed is one, since high operating speeds will positively influence the efficiency of work in the winery, but is negative when up to 3 mg/L of dissolved oxygen can be contributed [9]. The choice of the pump used is another important factor in oxygen intake, the peristaltic pump being the one with the lowest intake of 0.12 mg/L, followed by the piston pump with a 0.2 mg/L intake and finally the centrifugal pump with the highest intake: 0.7 mg/L of dissolved oxygen [7]. In filtration, the addition depends on the type of filtration used, so with a rotary vacuum filter, the levels are 2.2 mg/L of dissolved oxygen [6], but in the case of the earth filter, the levels depend very much on the specific characteristics of each process. Previous works established O_2_ addition levels between 0.1 and 0.7 mg/L [9]; 0.25 mg/L [5]; or 0.157 mg/L [6]. Values for the plate filter ranged from 0.315 mg/L [6] to 0.45 mg/L of dissolved oxygen [9]. Membrane filter work reported oxygen addition levels in the order of 0.075 mg/L [5]; 0.147 mg/L [6]; 0.2 mg/L [9] or 0.33 to 0.69 mg/L dissolved oxygen [12]. Finally values for tangential filtration were between 0.21 and 0.26 mg/L [5,6,12]. In cold tartaric stabilization, temperature is very important because temperatures of −5 °C can be reached, exponentially increasing the solubility of oxygen. Thus, two types can be differentiated: static tartaric stabilization with a contribution of 1.26 mg/L and continuous tartaric stabilization can provide up to 4.1 mg/L of dissolved oxygen [5].

Finally, when bottling, it is recommended to maintain adequate levels of FSO_2_. This protects the wine and preserves it without deteriorating its organoleptic characteristics throughout its useful life in the bottle, but without exceeding the levels as this would cause irritating and unpleasant odors for the consumer. It has been possible to observe that there is a wide range of oxygen supply in the bottling process, since it depends on several factors such as the temperature of the wine, the inerting and purging conditions at the beginning of the process, the total or partial emptying of the tank that supplies the bottling line. It thus ranges from 0.23 to 3.87 mg/L of dissolved oxygen, with the average supply value being 1.60 mg/L [8].

There are many studies that have established the contributions of each operation in the winery to the musts or wines; however, one part of all these operations has hardly been taken into account until now. There are no studies on the different materials of hoses which are used in any operation that requires moving the wine, with the exception of wineries that do everything by fixed stainless steel pipelines or those that move the wine by gravity or with small tanks that can be moved by crane. In addition, when using any equipment, it is essential to use connectors, all requiring the use of gaskets that guarantee a correct liquid tight closure. These gaskets of different materials can be found on the market and there are no clear recommendations as to which materials are the most adequate to avoid addition of atmospheric oxygen. In this work, different inert gases were also studied, both for hoses and tanks. All this produced a series of characterizations of the O_2_ addition to wine in its tank-to-tank racking in a winery considering different points: the material of the hoses, the connectors and their joints, the inert gases used to flush hoses and empty tanks and to blanket the full tanks. Finally, recommendations have been established to ensure that O_2_ addition is kept to a minimum.

## 2. Materials and Methods

### 2.1. Dissolved Oxygen Measurement

To measure oxygen, TROXROB10 (Pyro-Science GmbH, Aachen, Germany) Trace Range Robust Oxygen probes (measurement range: 0–10% O_2_; limit of detection: 0.004% O_2_) were used to perform high-precision measurements with values close to 0% O_2_ both in gas and in dissolved form, in conjunction with a FireStingO_2_ optical oxygen measurement device (Pyro-Science GmbH, Germany). For the measurements carried out in the winery, 10 m long Oxygen Dipping Probes DP-PSt6, accurate to ±1 ppb or ±3% of the respective concentration, were used connected to two OXY-4 trace-measuring equipment (PreSens GmbH, Germany). All equipment was periodically calibrated at two points according to the manufacturers’ instructions using a GM-3 gas mixer (Sensor Sense, Nijmegen, The Netherlands).

### 2.2. Model Wine

When determining the combined effect that a type of hose and connection has on the racked wine is necessary and in order to observe the evolution of oxygen levels, it is important to guarantee the absence of oxygen-consuming compounds in the wine. For this purpose, a synthetic wine based on a hydroalcoholic solution with characteristics similar to those of wine was used. This distillate, which is diluted to 12.5% *v/v*, has been proven not to consume oxygen. For de-gassing model wine to low oxygen levels, two membrane contactors, Liqui-Cel^®^ 4 × 13 Extra-flow modules (3M, Maplewood, Ramsey, MN, USA), were used in series mode. The Liqui-Cel^®^ modules were operated in the so-called transverse-flow for the liquid, meaning that model wine flowed on the shell side of the membrane module. Inside the hollow-fiber membranes (tube or lumen side of the module), a low pressure of P_a_ = 60 mbar (vacuum) was maintained and nitrogen was supplied as a stripping gas working together in combo mode at a flow rate of 10 L/min.

### 2.3. Hoses

The hoses made with Butyl Rubber (IIR) are those most widely recommended for the movement of wine in wineries, so this material has been the most examined and therefore used as a reference in this work. Diameters DN32 and DN50, the most common in small and medium wineries habitually using hoses for wine racking, and the effect of the length of the hose made of this material were studied. Hoses made of other materials, such as Nitrile Butadiene Rubber (NBR), Ethylene Propylene Diene Monomer rubber (EPDM), Ultra-high-molecular-weight polyethylene (UHMW) and Natural Rubber (NR), were also analyzed (Table 1).

#### 2.3.1. Butyl Rubber

It is an elastomeric copolymer of isobutylene with small amounts of isoprene (1–2.5% in moles). Its most important property is its very low permeability to gases and liquids. The Vinoflex easy (IVG Colbachini, Padova, Italy), suitable for suction and discharge of non-fatty liquid food in diameters DN32 and DN50, was studied.

#### 2.3.2. Nitrile Butadiene Rubber

The DN32 hose used in the study was Igienoil-HF 10 S7B (Tubi Thor, Lesmo, Italy). It is used for the suction and supply of milk, yogurt, dairy products, fatty foods of vegetable origin, fruit juices and low alcohol drinks. The interior of the hose is a white-colored food grade NBR rubber tube totally devoid of odor and taste and has a specular appearance. The tested DN50 hose was the Millenium easy (IVG Colbachini, Padua, Italy): it is a hard wall corrugated hose for suction and supply of many food products such as olive oil, wine, beer, fruit juice, fatty food, milk and alcohol up to 96°. It is a white, NBR, smooth, food grade synthetic rubber tube, tasteless and odorless. 

#### 2.3.3. Ethylene Propylene Diene Monomer Rubber

EPDM rubber is a sulfur vulcanizable thermoset elastomer produced from ethylene, propylene and a small amount of unconjugated diene such as hexadiene. The DN32 hose tested was the Roiman AM410E, currently LM1S (Semperit, Wimpassing, Austria). It is a flexible suction and discharge hose for use in breweries and food and beverage industries, suitable for use with alcohol (max. 40%), soft drinks, fatty foods (max. 36%) and non-fatty foods. The DN50 hose studied was the Milk Service (IVG Colbachini, Padova, Italy).

#### 2.3.4. Ultra-High-Molecular-Weight Polyethylene

Very-high-molecular-weight cross-linked polyethylene is a linear thermoplastic polymer of ethylene with a molecular weight of millions. It has good wear and chemical resistance, hardness and anti-friction properties, but poor processability. Processed by compression molding and water hammer extrusion, the 32 mm hose tested was the Superior-HF SBB Blue (Tubi Thor, Lesmo, Italy). The DN50 hose tested was the Shetland (IVG Colbachini, Padova, Italy): a hard wall hose for suction and delivery of alcohols up to 96° without altering their smell or taste.

#### 2.3.5. Natural Rubber

Natural rubber is polyisoprene. Both chemical and environmental resistance and mechanical properties are improved by cross-linking (vulcanization), usually by sulfur treatment. Air permeability varies from 11.8 × 10^−8^ at 40 °C up to 43.9 × 10^−8^ at 80 °C, expressed in cm^3^·cm/cm^2^·s·atm; in studies with nitrogen, the permeability at 21.1 °C is 6.12 × 10^−8^ cm^2^/s·atm. The tested DN50 hose was the Vinoflex NR (IVG Colbachini, Padova, Italy), natural rubber, only available in a 50 mm diameter. It is the same as the butyl hose, but the material is NR, natural rubber.

### 2.4. Connections

The two most commonly used quick connectors in oenology are the threaded ones, which consist of a threaded male and a female with a threaded nut inside to guarantee watertightness by means of a gasket (DIN 11851, SMS, Male-type joints, etc.), and the quick connectors without threads that also incorporate a gasket to ensure impermeability to liquids (CLAMP, Spherical type joints, etc.).

**Figure 1:**
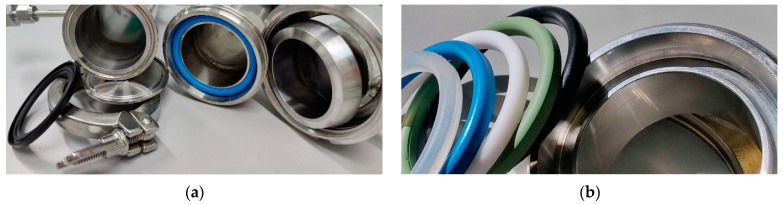
(**a**) Detail of the Clamp connection (Tri-clover-Tri-clamp) on the left versus DIN 11851 on the right; (**b**) Seals for DIN 11851 DN50 connection of different materials: Q/VMQ, NBR, PTFE, FKM/FPM and EPDM (left to right).

In this work, the two most common connections were tested: the DIN 11851 connection with thread and the Clamp type connections (ISO 2852, DIN 32,676 and BS 4825-3) with fast coupling, also known as Tri-clamp or Tri-clover. For each of these, gaskets of different materials marketed in both types of connection were studied. The gaskets most commonly supplied with the fittings are made of NBR, Nitrile rubber (acrylonitrile butadiene copolymer), although gaskets of EPDM, Fluorocarbon (FKM/FPM) also known as Viton^®^, silicone rubber (Q/VMQ) and Polytetrafluoroethylene known as Teflon (PTFE) are available (Figure 1). The diameters studied were DN32 (32 mm), DN50 (50 mm) and DN80 (80 mm).

### 2.5. Inert Gases

#### 2.5.1. Nitrogen (N_2_)

Nitrogen has a density of 1.1694 g/L at 1 atm. and 15 °C, lower than that of air 1.225 g/L under the same conditions (0.97%). Its bacteriostatic power is very limited and its capacity to inhibit the action of oxygen is reduced. In this work, N_2_ was used in 50 L bottles (Carburos Metálicos, Air Products Group, Barcelona, Spain).

#### 2.5.2. Carbon Dioxide (CO_2_)

Carbon dioxide is a colorless gas, its density is 1.84 g/L, at 1 atm. and 15 °C and is higher than that of air 1.225 g/L under the same conditions. CO_2_ is a much more soluble gas than nitrogen, being dependent on pressure and temperature, so during its use in wine, this property must be taken into account. The degree of solubility for saturation of CO_2_ in a wine stabilized at 20 °C and 1 atm. is 1.6 to 1.69 g/L of CO_2_. These values may increase if working at a lower temperature and higher pressure. The food grade CO_2_ was supplied in 50 L bottles (Carburos Metálicos, Air Products Group, Barcelona, Spain).

#### 2.5.3. Argon (Ar)

It is a monoatomic inert gas, its density is 1.669 g/L at 1 atm. and 15 °C, is higher than that of air 1.225 g/L under the same conditions. The Ar was supplied in 50 L bottles (Carburos Metálicos, Air Products Group, Barcelona, Spain).

#### 2.5.4. Mixture of Nitrogen and Carbon Dioxide (N_2_ + CO_2_)

Mixture of gases in a percentage of 80% vol. nitrogen and 20% vol. carbon dioxide: in its gaseous state, it has a density of 1.3 g/L, so is heavier than atmospheric air. This gaseous mixture was supplied in 50 L bottles (Carburos Metálicos, Air Products Group, Barcelona, Spain).

#### 2.5.5. Mixture of Argon and Carbon Dioxide (Ar + CO_2_)

Mixture of gases in a percentage of 80% vol. argon and 20% vol. carbon dioxide, with a density in the gaseous state of 1.41 g/L, higher than that of atmospheric air. This gaseous mixture was supplied in 50 L bottles (Carburos Metálicos, Air Products Group, Barcelona, Spain).

### 2.6. Measurement Protocols

#### 2.6.1. Oxygen Permeability Measurement of Hoses and Connectors

To measure the oxygen permeability of hoses, DIN 11851 fittings with NBR seals were used in all cases, as these are usually supplied with the fittings. With this type of connector, a manifold was manufactured to control pressure and temperature and also a valve for evacuating the oxygen by cycling CO_2_ and N_2_. To ensure the absence of oxygen, the CO_2_ was injected through a tube in the middle of the hose; this was placed with the outlet of the valve upwards so that the hose was filled with CO_2_ by density and air came out via the space left in the valve by the tube. Next, N_2_ was injected with the fitting facing downwards to remove the previous CO_2_.

**Figure 2:**
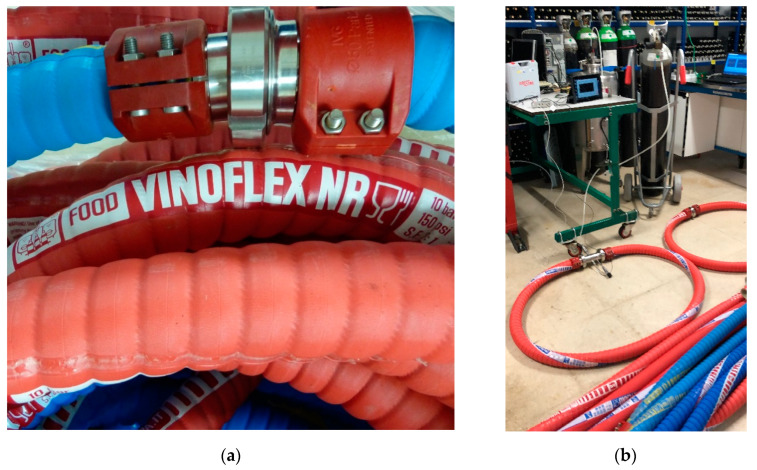
(**a**) Detail of the hoses with the connector DIN 11851; (**b**) View of the characterization ring of the hoses of different materials.

The hoses were tested in a three-meter section that is equivalent to a fluid volume of 2.41 L in DN32 and 5.89 L in DN50 (Figure 2). The results were corrected by eliminating the oxygen inlet produced by the double fitting, thus obtaining the oxygen inlet rate (Oxygen Transmission Rate; OTR) in that hose section, which characterized the oxygen permeability of the hoses made of different materials. IIR, EPDM, UHMW and NBR from different producers were studied for DN32, and IIR, EPDM, NR and NBR, from the same producer (IGV Golbachini Spa, Padua, Italy), were tested for DN50.

**Figure 3:**
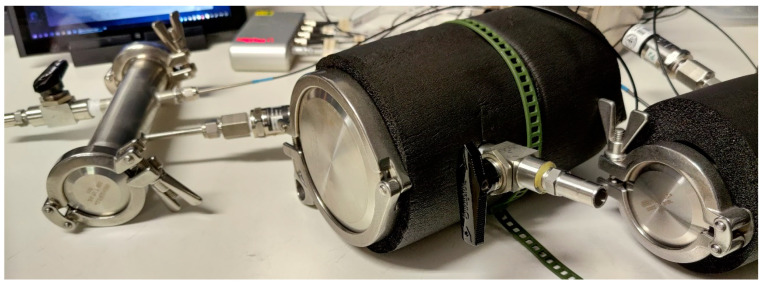
Detail of the three sizes of tested Clamp connectors (DN32, DN50 and DN80) with both blind covers and where the OTR was tested for two gaskets. OTR: Oxygen Transmission Rate.

The same procedure was used to measure the gaskets of different materials but replacing the hose section with two blind caps (Figure 3) that allowed the measurement of atmospheric oxygen infiltration in the two types of fittings, Clamp and DIN, in three sizes (DN32, DN50 and DN80) and with gaskets of different materials (Q/VMQ, NBR, PTFE, FKM/FPM and EPDM). 

#### 2.6.2. Hose Flushing with Inert Gases

To carry out the flushing measurements of the hoses with the different inert gases, the consumption of each of the gases tested in volume was studied, for which a variable area flowmeter calibrated for air with a range between 2 and 25 L/min was used, model Rate-MasterR series RM-22 (Dwyer Instruments, Inc. Michigan, IN, United States). In order to compare the actual volumetric flow rate of each gas in relation to the measured air flow rate, the specific constant of each gas was used according to (1):

The values being q_air_ (L/min) and R_gas_ (J/kg·K): air = 287.042; N_2_ = 296.786; CO_2_ = 188.920; Ar = 208.130; N_2_/CO_2_ = 275.213 and Ar/CO_2_ = 204.288. With these corrections, the flow conversion factor for each gas was: N_2_ = 1.01683; CO_2_ = 0.81127; Ar = 0.85152; N_2_/CO_2_ = 0.97918 and Ar/CO_2_ = 0.84362.

#### 2.6.3. Tank Flushing with Inert Gases

To carry out the measurements in winery operations with the different gases, 1000 L and 1500 L stainless steel tanks with cooling jackets on the upper part of the tank were used. Two different protocols were followed, one to measure the effect of tank air purging on the wine, and the other to measure the effect and persistence of the blanket of inert gas over the wine in both the emptied tank and the filled tank.

- Gas Phase Protocol

**Figure 4:**
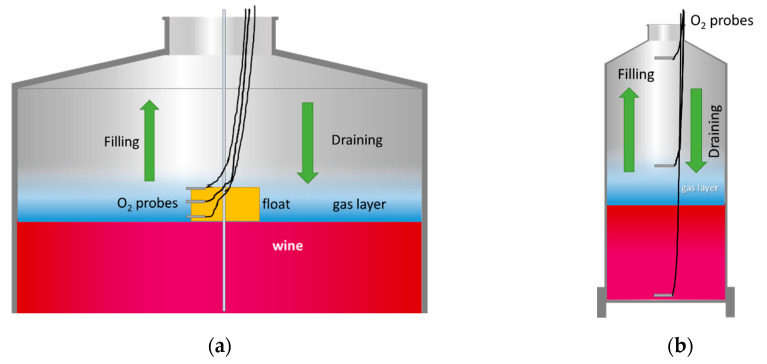
(**a**) Scheme of the float as well as the oxygen dipping probes at different constant distances from the liquid layer in gas mode; (**b**) Situation of the oxygen dipping probes located at three fixed heights in the liquid mode.

The generation and persistence of the protective layer of inert gas on the surface of the model wine was investigated using this protocol. For this purpose, Oxygen Dipping Probes DP-PSt6 (PreSens GmbH, Germany), accurate to ±1 ppb or ±3% of the respective concentration, were placed on floats at different distances from the liquid surface in both tanks: 2, 10 and 20 cm in the tank to be filled and 1, 2.5 and 5 cm in the tank to be emptied (Figure 4a). The floats kept the probes always close to the liquid, allowing the efficiency and persistence of the different inert gases to be checked. The oxygen drop produced during the inertization of the empty tank (tank flushing; purging) was measured. In addition, the oxygen content of the blanket in contact with wine was measured during racking, both in the tank being filled (purging evolution) and in the tank being emptied (blanket evolution) at the mentioned heights.

- Liquid Phase Protocol

The effectiveness of inerting was investigated by measuring the dissolved oxygen in the model wine using this protocol. For this purpose, Oxygen Dipping Probes DP-PSt6 (PreSens GmbH, Germany) were placed at three different heights of the tank: at the top, middle and bottom of both the target and source tanks (Figure 4b). 

During the tests, the same procedure was used for each type of inert gas analyzed, with three tests being carried out for each one. The empty tank was flushed with an inert gas and filled with model wine, or the filled tank was blanketed and emptied of model wine, or both situations at the same time. In the air-purged empty tank situation, one third of the volume of the tank inert gas was injected. In the full tank, inert gas was injected to generate a blanket of 5 cm thickness over the liquid surface. A flexible impeller pump model EP MIDEX (Liverani s.r.l.; Ravenna, Italy) was used for the model wine racking.

### 2.7. Statistical Analysis

To study the statistically significant differences according to the studied parameters, a one one-way analysis of variance (ANOVA) at the 95%, 99% or 99.9% probability level according to Fisher’s least significant difference (LSD) were determined. Statistical analysis was carried out using the STATGRAPHICS Centurion 18 program (Statgraphics Technologies Inc., The Plains, VA, USA).

## 3. Results

### 3.1. Connections

The O_2_ permeation was measured with the different connectors in triplicate and express the variation of the partial pressure of the O_2_ with time for two connectors with their respective gaskets. Table 2 shows the results for the two types of connectors, in the three most common diameters in the wineries and with the gaskets of the five materials usually available. The results of the analysis of variance are also shown. It is important to note that in each brand name, the colors of the gaskets correspond to different materials, that is, the color should not be taken as an indication of the gasket material.

### 3.2. Hose Permeation

Regarding the analysis of the atmospheric oxygen permeability of hoses made of different materials, in each case the effect of the connector and the seal used in the analysis was eliminated. The tests were carried out in triplicate on rings of 3 m hose lengths, as described in the Materials and Methods section.

As can be seen in Table 3, in order to offer the value per meter of hose, the original measurement was modified, correcting the effect of the connectors beforehand. In order to be able to compare the effect it would have on the liquid regardless of the diameter, these values are offered per liter. 

### 3.3. Hose Purging

**Figure 5:**
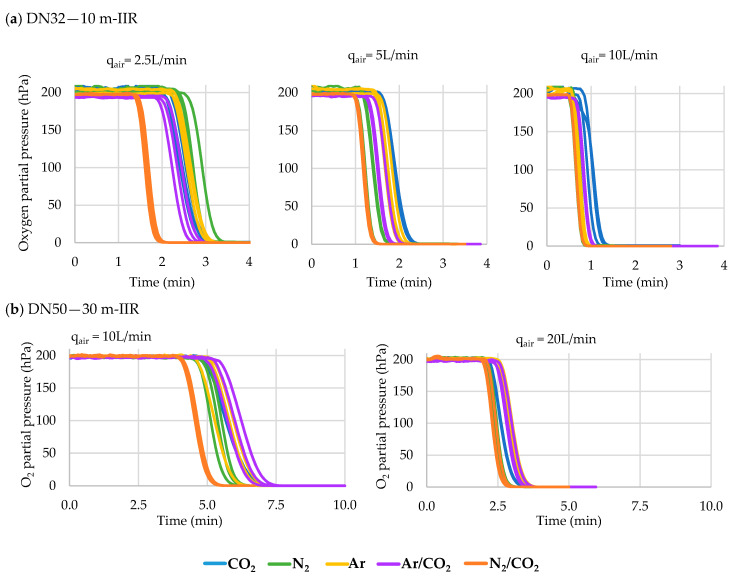
Time needed to purge the air from the interior of IIR hoses with different inert gases at different flow rates (10 L/min): (**a**) DN32 and 10 m length; (**b**) DN50 and 30 m length. The remaining measurements of the other hose materials are available in the Supplementary materials Figures S1 and S2.

This section presents the data obtained for the air purging of oxygen from the interior of hoses made of different materials thanks to the five inert gases commonly used in oenology. Up to three different flow rates (measured in air flow rate) of each gas were tested in triplicate and later corrected to know the real flow rate spent for each gas (see Section 2.6.2). The lengths tested were 10 m for the DN32 hoses with an internal volume of 8.04 L, while for the DN50 hoses the tests were carried out with 30 m length hoses with an internal volume of 58.90 L. Three different flow rates of each of the five inert gases tested for each of the four materials in the case of the DN32 diameter (180 tests) and only two gas flow rates in the case of DN50 diameter (120 tests) were carried out in triplicate (Figure 5). 

The data indicate that the DN32 diameter, when using IIR hoses, required significantly less time with N_2_/CO_2_ for 2.5, 5 and 10 L/min flow rates. The same results were obtained using hoses of the other materials (UHMW, EPDM), except for a 10 L/h flow rate, which resulted in there being no difference between the different gases with an NBR hose. In the case of using DN50 hoses, the data indicate that the higher the flow, the shorter the time needed to lower the oxygen partial pressure. When using IIR and NBR hoses, the inertization time required was similar for all gases with 10 L/min flow, while the time required was significantly lower with the use of N_2_ or N_2_/CO_2_ when using a 20 L/min flow. When the hose was made of UHMW or EPDM, the time needed was significantly less with the use of N_2_ at both flows but only with a 10 L/min flow with an NR hose. Furthermore, all data indicate that the higher the flow rate, the shorter the time needed for inertization, regardless of the hose and gas used (Table 4). 

#### The Effect of Hose Length

To study the effect that the length of the hose, and therefore the interior volume to be inerted, could have on the time used for inerting, a 50 m long DN32 butyl hose was measured. Once tested, it was cut into two pieces of 20 m and 30 m, respectively, and finally a 10 m piece was analyzed. Table 5 shows the data obtained and the ANOVA results.

The results indicated that at a flow rate of 5 L/min, the shortest purging time was achieved with N_2_ or N_2_/CO_2_ for all the hose lengths studied and also Ar for more than 10 m. In the case of a flow rate of 10 L/min, the results were similar, improving the results with the use of Ar with a hose longer than 10 m. Finally, at 20 L/min, the shortest purging time was achieved with N_2_ or N_2_/CO_2_ or Ar for 10 and 30 m length hoses.

### 3.4. Racking Tank to Tank

**Figure 6:**
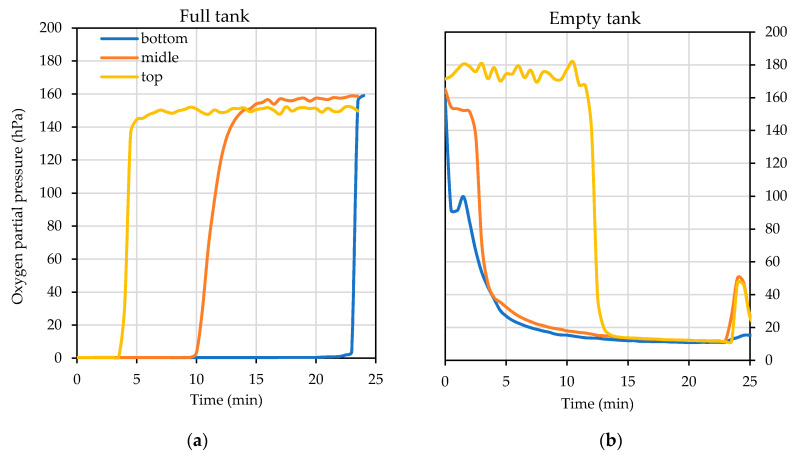
Evolution of the dissolved oxygen level at different points in the tanks that are emptied (full tank, (**a**)) and filled (empty tank, (**b**)) during a transfer with both connections at the bottom of the tanks.

These tests were carried out with model wine (see Materials and Methods section) and therefore the measurements were done with submersible probes measuring the partial pressure of dissolved oxygen in the liquid. The advantage of the probes used is that they can measure both in the liquid and in the gas. Before carrying out the purge tests in two 1500 L tanks and in two 1000 L tanks involved in wine racking, numerous measurements were carried out beforehand which yielded oxygen increases in the model wine of 15.03 ± 0.15 hPa in the larger volume tanks and of 12.21 ± 0.57 hPa in the smaller ones. These values were taken as a reference to compare the different results obtained in the different types of inerting (Figure 6).

#### 3.4.1. Tank Purging and Blanketing 

In these tests, the effect of flushing with inert gases was studied in the tank to which the wine was transferred (empty tank). To do this, all the oxygen was displaced from a layer of sufficient thickness to avoid its addition to the wine filling the tank. In addition, the effect of blanketing the tank that was being emptied (full tank) was studied, with a blanket of gas to prevent O_2_ addition to the wine with the air intake when the tank was emptied. Finally, the test was repeated with both processes in both tanks. The three options were tested with the different inert gases used throughout the study and are included in Table 6. As a rule, a layer was formed with a thickness of 1/3 of the height of the tank.

#### 3.4.2. Efficiency and Intensity in the Use of Inert Gases

**Figure 7:**
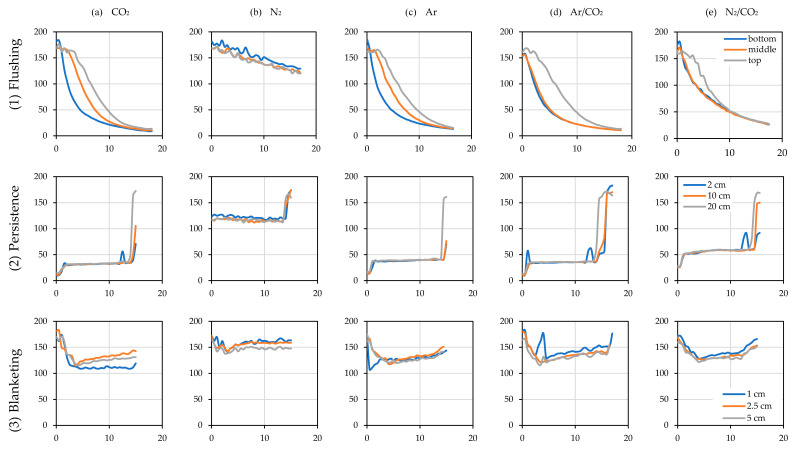
Evolution of the oxygen partial pressure (hPa, Y axis) with time (min, X axis) in the empty tanks that were filled (**1**) and (**2**) and in the full one that was emptied (**3**).

During the process of flushing, wine filling (Persistence) and wine emptying (Blanketing) of the racked tanks, the placement of probes in the gaseous layer next to the surface of the wine in the floats described in the Materials and Methods (Section 2) allowed the efficiency in the reduction of the oxygen level to be known (Figure 7).

Blanketing indicates the capacity of the layer of inert gas injected above the surface of the wine to avoid addition of oxygen from the atmospheric air entering the tank during emptying or leaving it during filling. The evolution of the partial pressure of oxygen over time in the empty tank by Flushing is shown in Figure 7(1) when the air was purged with an inert gas using one third of the tank volume and with a flow rate of 20 L/min in air calibrated rotameter of the different gases tested with the measuring points at the bottom, the middle and the top of the tank (Figure 4b). Persistence describes the evolution in pO_2_ of the blanket at 2, 10 and 20 cm above the surface of the wine filling the empty tank (Figure 4a) previously purged when it starts to fill through the bottom valve (Figure 7(2)). Finally, Blanketing shows the pO_2_ at 1, 2.5 and 5 cm above the surface of the wine being transferred in the tank being emptied at different times when enough gas volume was injected to form a 5 cm blanket and with a flow rate of 10 L/min to avoid turbulence above the wine surface (Figure 7(3)).

The level of inert gas mixture replacing wine ullage was evaluated with the inert gas that offered the best results, Ar in the 1500 L tank and CO_2_ in the 1000 L tank. Each tank was purged with a volume of inert gas one-half, one-third or one-quarter of its volume. The results obtained are shown in Table 7 and represent the value of wine O_2_ addition when filling a purged tank with different volumes of each inert gas tested.

#### 3.4.3. Impact of Wine Transfer at Different Stages of Inert Gas Uses

To discover the influence of different intensities in the use of inert gases on wine racking in the winery, it was decided to carry out a series of type tests in 1500 L tanks using model wine and a combination of the different tests carried out previously. Butyl hoses DN32 were used as they obtained better results avoiding oxygen addition, together with DIN 11851 connectors. A flexible impeller pump described in Section 2.6.3 was used for the model wine racking. CO_2_ was used for tank purging and N_2_ for hose purging. 

**Figure 8:**
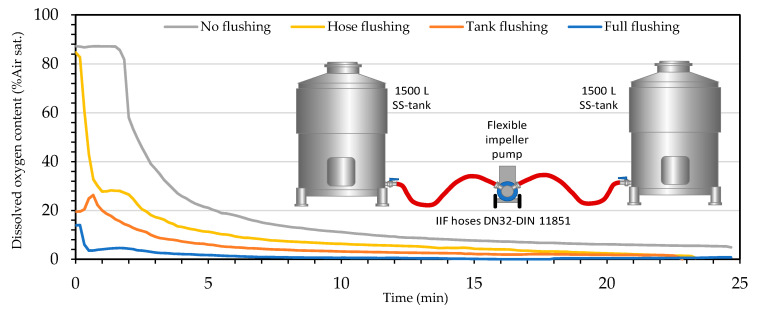
Evolution of the level of dissolved oxygen in wine after tank-to-tank racking with both tank intakes at the bottom and using different intensities of inert gases.

The results show the dissolved oxygen content of the model wine in the target tank during racking for the different situations tested (Figure 8; Table 8). First, the test was performed without the use of inert gases, then N_2_ was used to purge the air from the hoses and racking was performed again. In a third test, CO_2_ was used to flush the empty tank. Finally, racking was repeated with the deoxygenated model wine together with the empty tank purged with CO_2_, the wine tank with N_2_ blanketing and the hoses purged with N_2_. 

## 4. Discussion

### 4.1. Connections

Analysis of the test results provided knowledge of oxygen infiltration when different types of connectors were used, as well as different gasket materials, showing that not all the materials behaved in a similar way. Regardless of the diameter of the connection and the seal material, Tri-clamp fittings allowed less oxygen to enter than DIN 11851 fittings (Table 2).

The results showed that for each type of connector and for each diameter, the seal material played a very important role, since differences in oxygen permeation of several orders of magnitude were obtained. Comparing the use of a DN32, DN50 or DN80 diameter when using DIN type connection and NBR seal indicated that with DN32 more oxygen (hPa/h) was dosed than with DN50 or DN80 (*p* = 0.0232). In the event of using FKM/FPM or PTFE seals, the results indicated that more oxygen was dosed with the DN80 diameter than with DN32 or DN50, (between which there were no differences: *p* = 0.0194 and 0.0361, respectively). The use of Q/VMQ gaskets showed that a greater quantity of oxygen was dosed with diameter DN80, followed by DN32 and the lowest infiltration was obtained with diameter DN50 (*p* = 0.0000). Regarding the use of EPDM gaskets, no statistically significant differences were found using the different diameters studied (*p* = 0.1821). The use of CLAMP type connections indicated that with NBR and EPDM gaskets there were no statistically significant differences between the diameters tested (*p* = 0.3316 and *p* = 0.2768, respectively); however, a greater quantity of oxygen was dosed with FKM/FPM gaskets and with the DN80 diameter than with DN32 or DN50, between which there were no differences (*p* = 0.0377). On the other hand, when using Q/VMQ gaskets, the largest infiltrations occurred with DN32, followed by DN50 and the smallest with DN80, these being statistically different (*p* = 0.000). Finally, it should be mentioned that the infiltration through PTFE joints was significantly different according to the diameter used (*p* = 0.000), with DN80 being higher, followed by DN50 and finally DN32.

Viton (FKM/FPM) was the material that allowed a more oxygen-tight connection, regardless of the diameter and for both types of connectors, as recorded in the literature [13,14,15,16,17]. If the variation of the oxygen partial pressure (OTR) with respect to Viton (FKM/FPM) is compared for each material, for each diameter and for each type of connector, a relative index is obtained for every case (Table 9). The NBR had a slightly worse behavior in DN32, while performance by DN50 and DN80 was similar. EPDM was similar in DN80, but in smaller diameters it offered transmission rates between 3 and 5 times higher than FKM/FPM. On the other hand, Q/VMQ silicone seals were worse, since, except for DN80, they showed between 80 and 1500 times higher infiltration rates. Finally, the bad results of the Teflon seals (PTFE) stood out, probably due to the disadvantage of its greater hardness, making it more difficult to achieve reliable metal-polymer-metal seals (Table 9).

**Figure 9:**
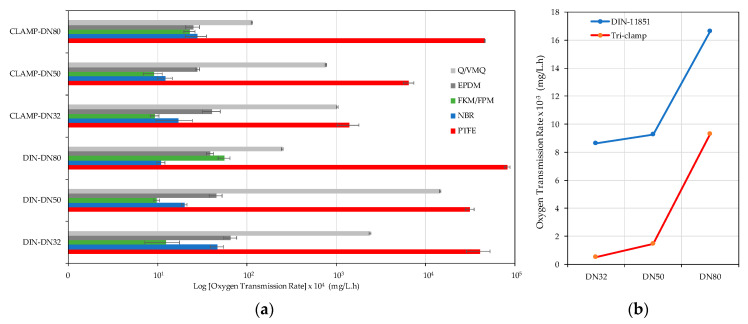
(**a**) Oxygen Transmission Rate (mg/L·h) in the different connectors according to diameter as well as the gasket material referred to the volume of the connector; (**b**) Comparison of mean-corrected OTR with the volume for both connectors according to diameter.

In order to evaluate the effect of all the factors studied (joint material, diameter and type of connector) on the volume affected by infiltration, the results were adapted to be expressed in mg/L·h, which allowed comparison of the overall behavior of the joint material for all the diameters of both types of connectors, considering the volume of each one (Figure 9a). For most diameters, FKM allowed a more tightly sealed connection, followed closely by NBR and EPDM. On the other hand, Q/VMQ is reported in the literature as a more permeable material than the rest of the elastomers tested. Although PTFE is the material with the lowest oxygen permeability [18,19], it is not very suitable for this type of connector due to its low elasticity preventing correct operation due to its continuous assembly and disassembly in the winery. Overall, it was found that the DIN type connection caused a higher O_2_ addition than the CLAMP (*p* = 0.0474). The biggest differences were found using Q/VMQ followed by PFTE and EPDM with *p* = 0.0039, *p* = 0.0068 and *p* = 0.0364, respectively. However, when NBR or FKM/FPM were used, there were no statistically significant differences between both with *p* = 0.2869 and *p* = 0.1474, respectively. Analyzing the use of these two materials with different diameters, with NBR and DIN connection, more O_2_ addition occurred at smaller diameters (*p* = 0.0002), while there were no significant differences between diameters if a CLAMP connection was used (0.0546). Regarding FKM/FPM, the results indicated that the highest O_2_ addition occurred with 80 mm both with DIN connection (*p* = 0.0001) and CLAMP (*p* = 0.0006).

If the behavior of both connectors is compared globally, it can be seen that, in general, the Tri-clamp connector resulted in more watertight joints than the DIN 11851 connector, regardless of the diameter or the material of the seal (Figure 9b).

### 4.2. Hose Permeation

**Figure 10:**
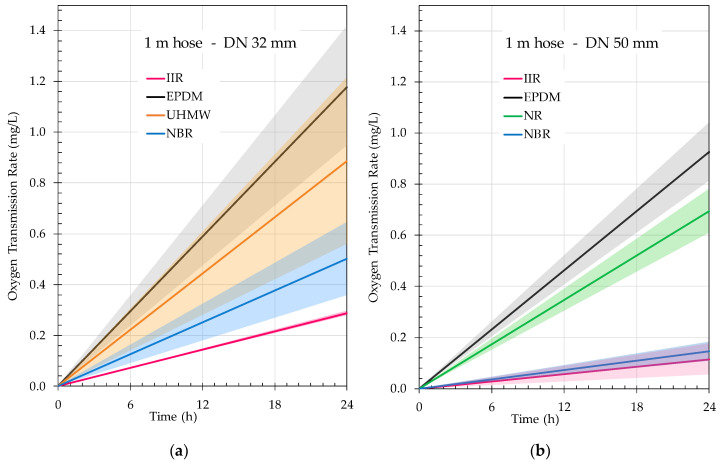
Comparison of OTR (mg/L·h) corrected with the volume hose for both diameters, (**a**) DN32 and (**b**) DN50; and for the different materials tested. Thin line color represents standard deviation from the mean of each material.

The results obtained from the hose permeability analysis were performed on 3 m-long hoses with DIN 11851 connectors. The influence of the connectors on the OTR characterization of the hoses was considered. Therefore, the data obtained were converted to mg/L·h considering the pressure of the measurement gas and the temperature of the test for each meter of hose of the different materials being studied. The results of the oxygen intake for each liter of internal volume of the hose allowed hoses of different nominal diameters to be compared. In order to express the amount of oxygen that would be incorporated into each liter of wine contained within the hose, it would be necessary to know the mass flow rate of the fluid circulating through the hose, as well as the solubility of the oxygen in the transported liquid. The comparison of the use of a 32 mm or 50 mm diameter when using a IIR, EPDM or NBR hose indicated that, in all cases, significantly more mg/L·h was dosed with the 32 mm hose than with the 50 mm one (*p* = 0.0082, *p* = 0.0452, *p* = 0.0009) (Figure 10). 

Figure 10 allows comparison of the different materials tested, although the exact value of oxygen incorporated into the wine transported through the hose was not known. It can be seen that IIR hoses (Vinoflex easy) are those most impermeable to oxygen in both DN32 and DN50. NBR hoses (DN32: Igienoil-HF 10 S7B and DN50: Millenium easy) performed similarly to IIR. The EPDM hoses, although suitable for food transport, are not the most recommended to avoid a possible atmospheric oxygen addition since they showed greater values of OTR for both diameters. The OTR data obtained for UHMW and NR hoses were somewhat lower than those for EPDM, but equally far short of the good performance of IIR and NBR. Considering the diameter, it can be stated that the ratio surface/volume for each meter made DN50 hoses more recommendable as they offered clearly lower atmospheric oxygen transmission values than DN32. Thus, a meter of IIR DN32 hose had an OTR (mg/L·h) two-and-a-half times higher than the DN50 hose, three-and-a-half times higher in the case of NBR and 27% higher in the case of EPDM.

### 4.3. Hose Purging

**Figure 11:**
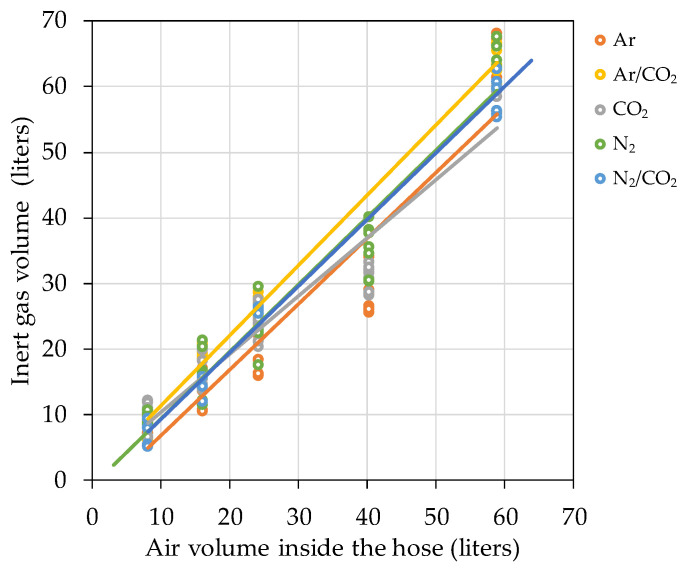
Inert gas volume consumed to reach a complete purge of the air volume inside the IIR hoses with CO_2_, Ar, N_2_/CO_2_, N_2_ and Ar/CO_2_.

The use of hoses is actually widespread in most wineries in all parts of the world. Although hose material is important to guarantee movement of the wine using the most appropriate material to avoid the inherent oxygen infiltrations, it is more important to ensure purging of the air they contain just before use. Although a priori it might be thought that evacuation of the air inside a hose should depend on the internal diameter and length, the results obtained allowed us to verify that the type of inert gas used, as well as the gas flow rate, are two determining factors when establishing the inerting time. The same levels of air purging were achieved for all the cases tested. However, the differences in the purging times need to be corrected with the real volumetric flows and with the cost of each gas in order to obtain a comparison of the expense involved in inerting hoses of different materials and diameters. The gas volume index (I_va/vg_), which is the ratio of the purged air volume and the inert gas volume used to displace the air, is normally used in these cases. When carrying out a complete purge with inert gas in the chemical industry, a minimum volume equal to 1.5 times the swept volume of the pipeline is recommended [20]. This type of index allowed comparison of hoses of different diameters and different lengths and collected the data of more than 400 tests carried out. Figure 11 shows the relationship between the volume of air displaced and the volume of inert gas used for IIR hoses of different lengths (10, 20, 30 and 50 m) and diameters (DN32, DN50).

Table 10 shows the index I_va/vg_ for each of the inert gases during purging of hoses of different materials. The index corresponds to the slope of the linear regression performed on all data from purging tests of hoses of each material and each of the inert gases. A perfect purge should be one in which the replacement of air in the hose with an inert gas is done entirely by displacement and only one volume of purge gas is required. Diluting or mixing the purge gas with the air in the hose will result in a greater amount of inert gas being spent on purging [21]. As can be seen from the results of the I_va/vg_ of the different gases, those gases with a higher specific gravity (CO_2_, Ar) are those that offer lower I_va/vg_, because they are capable of performing a displacement purging while barely mixing with the air inside the hose. However, those gases with a lower specific gravity required a greater volume of inert gas to sweep the air. The behavior of those gases differed from one hose to another depending on the material with which the inner layer of the hose was constructed. N_2_ stood out as a purging gas for EPDM and UHMW hoses with I_va/vg_ ≤ 1. Obviously, the volume of gas consumed is important, but the economic value of the gas is equally important. Table 10 includes the price (€/L) of the main inert gases for oenological use in Spain (Carburos Metálicos, Air Products, Barcelona, Spain) and shows that for all hoses the most economical gas was CO_2_. Although it is not a recommended gas for blanketing tanks of all types of wine due to its high solubility, in the case of its use for hose purging, it is more than justified, as the volume of wine in contact with the gas will be very small. N_2_ is the second-cheapest inert gas for purging air from hoses, although it costs twice as much as CO_2_ in most cases. The rest of the gases should be discarded, since they cost between three and four times higher than CO_2_ and would only be recommended for special operations: for example the blanketing of wines with dissolved carbon dioxide, when the necessary gaseous mixture would have to be determined in order not to alter its concentration in CO_2_ according to Lonvaud-Funel curves on dissolved CO_2_ versus CO_2_ headspace concentration [22].

**Figure 12:**
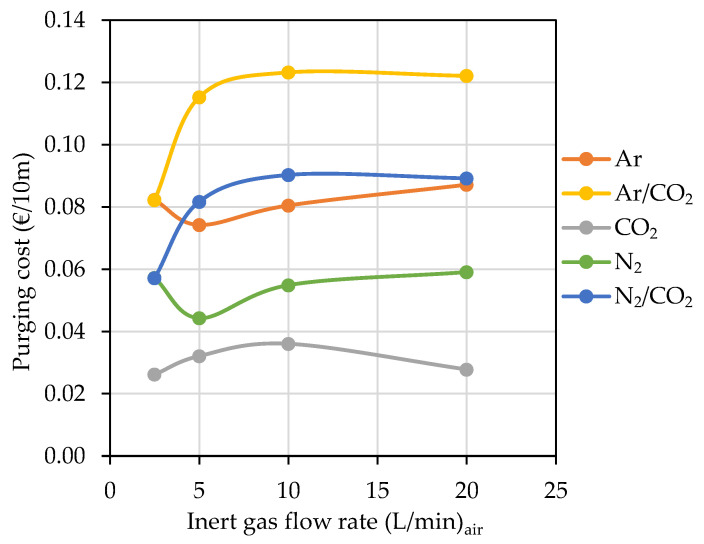
Comparison of inerting cost (€) for 10 m length hose of IIR with the tested inert gases at a different flow rate.

Although in general it might seem that the higher the flow rate, the shorter the purging time and therefore the lower the cost of inerting, other factors derived from the flow rate such as the speed of the gas which in a small section, such as in hoses, can cause turbulence, make it interesting to study this point. Gases with CO_2_, and therefore with a higher specific gravity, are those which allow displacement purging at low gas velocities, while N_2_ needs more flow to generate greater turbulence and compensate for its low specific gravity since it normally performs dilution purging. A separate case is Ar, because it behaves similarly to N_2_ in spite of its specific gravity. Analysis of the effect that the flow rate had on the cost associated with the inertization of 10 m of DN32 IIR hose, for each of the gases studied, is shown in Figure 12. It can be seen that globally the lowest cost is by using CO_2_ and the highest with Ar/CO_2_. It is interesting to note that the lowest cost with CO_2_, Ar/CO_2_ or N_2_/CO_2_ gases was produced with a lower flow rate, 2.5 L/min, which can be explained by the low turbulence produced when injecting the gas into the hose, and logically differed for each gas depending on its density and ability to mix (dilution purging) or displace air (displacement purging). Thus, when the flow rate was increased, there was a significant increase in cost with no statistically significant difference between using 5, 10 or 20 L/min. The advantage of using very low flows is to avoid the turbulence that highlights the advantage of CO_2_ in displacing the air. When the hose was purged with N_2_ or Ar, the lowest cost was by using a flow of 5 L/min, and there was no significant difference between the cost associated to its use at 2.5, 10 or 20 L/min. 

### 4.4. Wine Racking and the Use of Inert Gases

The practice of tank blanketing and flushing is a necessary operation to avoid the O_2_ addition produced when air enters a tank full of wine, or when it is emptied of wine and an empty tank is filled. The test allowed us to discover not only the influence of each of these operations, but also to determine which of the gases tested was suitable for optimizing the process of wine transfer with the lowest atmospheric oxygen addition. The tests, due to their complexity, were not carried out with repetitions, so the results are merely qualitative, not quantitative. However, they allowed us to visualize the effectiveness of the purging of the tank to be filled and the use of different gases to avoid atmospheric oxygen addition when wine was transferred tank-to-tank in the winery. In Figure 7, the graphs in the first row (1) Flushing show the evolution of the O_2_ partial pressure when the tank was purged by the top lid with inert gas one-third of the volume of the tank. The advantage of heavier-than-air gases (specific gravity > 1) is in their ability to displace air from the bottom of the tank, even though they are injected through the top lid. Gases capable of displacing air perform a displacement purging, while those which are not capable, such as N_2_, perform a dilution purging [23]. In a dilution purging, to lower 21% of O_2_ to 1% it is necessary to use three times the volume of the tank to purge it of inert gas, while in displacement purging, at least the volume of the tank is recommended [24] (p. 331). Thus, all gases except N_2_ were able to displace first the O_2_ in the lower part of the tank (blue line) and quite quickly even started to decrease the oxygen concentration in the upper part of the tank, performing in accordance with data obtained by the Institut National de la Recherche Agronomique (INRA, France) in 1996 [25], who compared the purging efficiency of the different gases and gas mixtures in terms of gas consumption per empty tank or headspace volume. During purging, all gases except N_2_ were able to lower the level of O_2_ to 2% (≈20 hPa) with the volume of gas injected. Although this is higher than the levels recommended by the chemical industry, it is an adequate level to consider the deposit free of atmospheric O_2_ in oenology [25]. It should also be noted that the N_2_/CO_2_ gas mixture was not able to reach the same pO_2_ levels as the rest of the inert gases tested, and therefore more time and thus more gas volume would be needed to reach the desired inerting levels of the empty tank, possibly because of the dilution and displacement purging being a mixture. The N_2_ in this case showed its limitations by diluting rather than displacing the air in the empty tank and only succeeded in lowering the partial pressure of oxygen a little.

Ar offered the best results, and other tests were carried out to discover the importance that the ratio “volume of inert gas used/volume of the tank to purge” could have in the final O_2_ addition to the wine that will fill the inerted tank. The results of using ½ and ¼ of the inert gas tank volume showed that, in the case of using one-third of the Ar tank volume, the increase in the final pO_2_ of the wine in the tank when filled was 6.7 hPa (325 µg/L if the solubility of O_2_ in water is considered), while when half of the tank volume was used, it was 3.9 hPa (188 µg/L), and when only a quarter of the Ar tank volume was used, the increase in pO_2_ was 7.2 hPa (347 µg/L).

Another interesting issue was the persistence of purging when the tank began to fill. The graphs in Figure 7(2), Persistence, show the evolution of pO_2_ at different distances from the upper surface of the wine filling the previously purged empty tank. In all tanks with very low oxygen levels (a, b, c and d), there was an initial strong rise when the wine first entered the tank and produced turbulence of the gaseous layer. Once the entry of the wine into the tank through the bottom valve was below the level of the wine, this turbulence was reduced and the gas layer, which prevents contact with air, remained fairly stable until the tank was filled, the probes on the float reached the neck of the tank and all the gas used in inerting was evacuated from the newly filled tank. N_2_ was not able to prevent O_2_ addition to the wine since the pO_2_ did not go down sufficiently during the purging phase beforehand. However, when filled with model wine, it was able to maintain the pO_2_ reached, so, although N_2_ showed consistency, it evidently did not guarantee the transfer of the wine without O_2_ addition with the volume of inert gas tested.

Finally, the ullage blanketing of a tank aims to prevent O_2_ addition in a full tank when wine is stored. In this test, the aim was to check whether this blanket prevented O_2_ addition when the tank was emptied of wine. Presumably the heavier-than-air gases could perform better than N2. The volume of gas injected was sufficient to form a layer of gas 5 cm above the surface of the wine. It must be remembered that the tank was full of wine up to the neck of the tank so the ullage was really small, and the blanketing was done a little before and during pumping of the wine into the empty tank. The unsatisfactory results obtained are shown in Figure 7(3), Blanketing. While CO_2_ allowed the levels of the blanket above the wine to be maintained with the lowest levels of oxygen, the rest of the gases, Ar, Ar/CO_2_ and N_2_/CO_2_ did this better than N_2_, which was again the worst performing gas for blanketing. These poor results may be due to the low height of the protective layer of 50 mm when some authors recommend 400 mm [25].

As for the tests carried out on tank-to-tank racking with model wine, it is interesting to note that the O_2_ addition levels of 0.56 mg/L of dissolved oxygen obtained without the use of inert gases were just over the moderate or low oxygen enrichments [6] and within the data reported in the literature [5,10]. If it is considered that 1.7 moles of SO_2_ react with 1 mol O_2_ [26,27], this type of racking under those conditions would suppose a consumption of 2.2 mg/L of FSO_2_. This simple operation would reduce the average FSO_2_ level of a protected pH 3.5 wine (9% in red wine with 25 mg/L and 5.6% in white wine with 40 mg/L). When inert gases are used in the flushing of hoses and tanks together with the blanketing of tanks with wine, tank-to-tank racking will reduce its impact to 0.2 and 0.1% of FSO_2,_ respectively.

## 5. Conclusions

For the first time, an exhaustive analysis of the rate of oxygen entry into the liquid (model wine) was carried out with the equipment used for racking in the winery, and it is clear that selecting the right materials is important. This would efficiently minimize atmospheric O_2_ addition and avoid the uncontrolled decrease of FSO_2_ in the wine.

Among the connectors studied, in general the Tri-clamp system performed better than the DIN 11851 system, regardless of diameter and joint material. 

The FKM/FPM gaskets sealed the wine from oxygen addition best with both types of connectors. NBR closely followed FKM/FPM, although with a O_2_ addition rate of at least double. The other materials performed far worse, acceptably in the case of EPDM, and the use of Q/VMQ and PTFE is not recommended. The latter stood out, since despite being the most impermeable material to O_2_ of those studied due to its low elasticity, it is not recommended for this type of connector due to its continued use.

As for hoses, of all those studied, IIR hoses proved to be the most impermeable to O_2_, although once again NBR was among the materials for hoses that were most impermeable to atmospheric O_2_. The rest of the hoses, except when considering other characteristics different from the product, did not guarantee the low O_2_ addition levels offered by those manufactured with IIR and NBR.

It is of interest that in order to reduce oxygen addition, if it is possible to choose, the impact of working with DN50 hoses is considerably lower than when working with DN32 ones.

Of the gases studied for hose purging, CO_2_ was the most effective for the recommended hoses as it had the lowest I_va/vg_ and consumed the least amount of gas, so it is the most economical, and if the winemaking process allows it. This gas clearly won if the economic factor was the most important. The N_2_ followed closely, although on average the operation cost twice as much. In EPDM and UHMW hoses, N_2_ performed better than CO_2_, but economically CO_2_ was clearly more advantageous.

The general comment accepted in the sector that Ar, in spite of being the most expensive gas, was the most interesting in hose purging due to its good performance, did not coincide with the results obtained in this work, since its economic cost was double that of N_2_ and four times more than that of CO_2_.

A very interesting result was that the most effective hose purging was that done by displacement, and it was better to use small flows that ensured low turbulence.

In tank purging, the importance of the specific gravity to obtain displacement purging became evident, and thus CO_2_ turned out to be the most effective gas followed by Ar. N_2_, when performing a dilution purging with the volume of gas over 1/3 of the tank volume, was only able to lower the O_2_ level of the deposits by a little. 

Consequently, if possible and for heavier-than-air gases, it is better to use inert gas in half and not only one-third of the volume of the tank to be purged, since O_2_ addition to model wine was reduced by half according to the results obtained with Ar.

Persistence of tank purging was adequately maintained for all gases, even for N_2_ at the levels reached during purging.

Blanketing of the tanks filled with 5 cm was not as successful as desired and it will probably be necessary to perform an injection equal to the volume of gas racked with the pump to ensure zero O_2_ addition to wine with the air entering the tank when it is emptied.

The widespread use of inert gases during the tank-to-tank racking process allows the operation to be carried out with hardly any oxygen addition, thus avoiding the consumption of FSO_2_ from the wine.

## Figures and Tables

**Table 1:** Compatibility of materials used in the manufacture of hoses and seals against different fluids.

Fluid	EPDM	NBR	Q/VMQ	FKM/FPM	PTFE	NR	UHMW	IIR
Wines and yeast	4	3	4	2–3	3–4	4	4	4
Concentrated fruit juices	1	1	1	3	3–4	4	4	4
Brewery	3–4	3	1–2	2–3	3–4	4	4	4
Alcohols	2–3	1–3	3–4	3–4	3–4	4	4	4
Ozone and Atmospheric Conditions	4	1–2	4	3–4	3–4	4	3	4

1 = Not suitable, 2 = Limited suitability level, 3 = Normal suitability level, 4 = High suitability level. (Source: Alfa Laval). EPDM: Ethylene Propylene Diene Monomer rubber; NBR: Nitrile Butadiene Rubber; Q/VMQ: silicone rubber; FKM/FPM: Fluorocarbon; PTFE: Polytetrafluoroethylene; NR: Natural rubber; UHMW: Ultra-high-molecular-weight polyethylene and IIR: Butyl Rubber.

**Table 2:** Oxygen permeation (×10^−3^ hPa/h) of seals made of different elastomers in different connectors and diameters (*n* = 3).

Connection Type	Diameter (mm)	Materials					
		NBR	FKM/FPM	Q/VMQ	EPDM	PTFE	*p* Level
DIN 11851	DN32	3.50 ± 0.57 ^c^	0.93 ± 0.39 ^a^	179.08 ± 4.13 ^b^	4.94 ± 0.83 ^a^	3046.83 ± 893.96 ^b^	0.0102 *
	DN50	1.50 ± 0.10 ^b^	0.73 ± 0.06 ^a^	1093.15 ± 21.02 ^c^	3.40 ± 0.56 ^a^	2366.80 ± 251.10 ^a^	0.0001 ***
	DN80	0.82 ± 0.08 ^a^	4.20 ± 0.66 ^b^	18.68 ± 0.48 ^a^	2.90 ± 0.26 ^a^	6194.63 ± 400.80 ^c^	0.0000 ***
Tri-clamp	DN32	1.28 ± 0.56 ^a^	0.70 ± 0.08 ^a^	76.70 ± 1.64 ^c^	3.06 ± 0.68 ^a^	105.25 ± 28.26 ^a^	0.005 **
	DN50	0.92 ± 0.18 ^b^	0.68 ± 0.16 ^b^	57.40 ± 1.02 ^b^	2.10 ± 0.10 ^a^	487.97 ± 67.26 ^b^	0.0003 ***
	DN80	2.08 ± 0.55 ^c^	1.72 ± 0.24 ^c^	8.53 ± 0.14 ^a^	1.88 ± 0.33 ^a^	3459 ± 41.21 ^c^	0.0000 ***

* *p* < 0.05 or ** *p* < 0.01 or *** *p* < 0.001 in rows indicates differences between the various materials for each connection and diameter. In columns different superscript letters indicate significant differences between diameter for the same connection and material. NBR: Nitrile Butadiene Rubber; FKM/FPM: Fluorocarbon; Q/VMQ: silicone rubber; EPDM: Ethylene Propylene Diene Monomer rubber and PTFE: Polytetrafluoroethylene.

**Table 3:** Oxygen transmission rate (hPa/h) achieved in hoses of different materials in the two tested diameters (*n* = 3).

Diameter (mm)	Material	OTR Measured (hPa/h)	OTR Hose ^(1,2)^ (hPa/h)	OTR Hose ^(1,2)^ (hPa/h·m)
DN32	IIR	0.0291 ± 0.0003 ^a^	0.0268 ± 0.00001 ^a^	0.0089 ± 0.00001 ^a^
	EPDM	0.1125 ± 0.0098 ^b^	0.1102 ± 0.0095 ^b^	0.0367 ± 0.0095 ^a^
	UHMW	0.0852 ± 0.0136 ^b^	0.0829 ± 0.0133 ^b^	0.0276 ± 0.0133 ^a^
	NBR	0.0493 ± 0.0060 ^a^	0.0470 ± 0.0057 ^a^	0.0157 ± 0.0057 ^a^
	*p* level	0.0093 **	0.0084 **	0.2508
DN50	IIR	0.0200 ± 0.0041 ^a^	0.0107 ± 0.0015 ^a^	0.0036 ± 0.0025 ^a^
	EPDM	0.0958 ± 0.0090 ^b^	0.0865 ± 0.0074 ^c^	0.0288 ± 0.0074 ^c^
	NR	0.0742 ± 0.0064 ^b^	0.0650 ± 0.0048 ^b^	0.0217 ± 0.0048 ^bc^
	NBR	0.0230 ± 0.0018 ^a^	0.0137 ± 0.0002 ^a^	0.0046 ± 0.0002 ^ab^
	*p* level	0.0019 **	0.0007 ***	0.0405 *

^1^: infiltration by the double DN32 connection was eliminated; ^2^: infiltration by the double DN50 connection was eliminated. * *p* < 0.05 or ** *p* < 0.01 or *** *p* < 0.001 in rows indicates differences between the various materials for each diameter, in columns different superscript letters indicate significant differences between material for the same diameter IIR: Butyl Rubber; EPDM: Ethylene Propylene Diene Monomer rubber; UHMW: Ultra-high-molecular-weight polyethylene and NBR: Nitrile Butadiene Rubber.

**Table 4:** Time required (min) to flush the hoses with the different inert gases and flow rates (*n* = 3).

Diameter	Material	Flow Rate	CO_2_	N_2_	Ar	Ar/CO_2_	N_2_/CO_2_	*p* Level
DN32 10 m	IIR	10	1.433 ± 0.076 ^a^	1.000 ± 0.050 ^a^	1.050 ± 0.000 ^a^	1.200 ± 0.000 ^a^	0.950 ± 0.000 ^a^	0.0000 ***
		5	2.533 ± 0.029 ^b^	1.830 ± 0.156 ^b^	2.300 ± 0.100 ^b^	2.067 ± 0.115 ^b^	1.617 ± 0.029 ^b^	0.0000 ***
		2.5	3.233 ± 0.076 ^c^	3.450 ± 0.132 ^c^	3.333 ± 0.058 ^c^	3.000 ± 0.150 ^c^	2.150 ± 0.050 ^c^	0.0000 ***
	NBR	10	1.283 ± 0.029 ^a^	1.117 ± 0.058 ^a^	0.767 ± 0.664 ^a^	1.200 ± 0.000 ^a^	1.000 ± 0.000 ^a^	0.0000 ***
		5	2.083 ± 0.029 ^b^	1.810 ± 0.053 ^b^	2.517 ± 0.029 ^b^	2.217 ± 0.076 ^b^	1.700 ± 0.000 ^b^	0.0000 ***
		2.5	3.150 ± 0.087 ^c^	2.383 ± 0.029 ^c^	4.017 ± 0.176 ^c^	3.067 ± 0.029 ^c^	1.883 ± 0.029 ^c^	0.3146
	UHMW	10	1.317 ± 0.029 ^a^	1.067 ± 0.029 ^a^	1.250 ± 0.000 ^a^	1.050 ± 0.000 ^a^	0.950 ± 0.000 ^a^	0.0000 ***
		5	2.053 ± 0.046 ^b^	1.667 ± 0.029 ^b^	2.083 ± 0.029 ^b^	2.067 ± 0.029 ^b^	1.633 ± 0.029 ^b^	0.0000 ***
		2.5	3.300 ± 0.050 ^c^	2.467 ± 0.029 ^c^	3.100 ± 0.087 ^c^	2.983 ± 0.029 ^c^	2.200 ± 0.000 ^c^	0.0000 ***
	EPDM	10	1.417 ± 0.029 ^a^	1.133 ± 0.029 ^a^	1.167 ± 0.029 ^a^	1.150 ± 0.000 ^a^	1.050 ± 0.000 ^a^	0.0000 ***
		5	2.313 ± 0.129 ^b^	1.890 ± 0.052 ^b^	2.367 ± 0.029 ^b^	2.400 ± 0.050 ^b^	1.717 ± 0.029 ^b^	0.0000 ***
		2.5	3.317 ± 0.076 ^c^	3.167 ± 0.275 ^c^	3.167 ± 0.029 ^c^	3.183 ± 0.076 ^c^	2.217 ± 0.029 ^c^	0.0000 ***
DN50 30 m	IIR	20	3.633 ± 0.029 ^a^	3.150 ± 0.087 ^a^	3.950 ± 0.050 ^a^	3.800 ± 0.132 ^a^	3.117 ± 0.076 ^a^	0.5211
		10	4.917 ± 4.258 ^b^	6.483 ± 0.176 ^b^	7.067 ± 0.416 ^b^	7.450 ± 0.300 ^b^	5.700 ± 0.050 ^b^	0.0000 ***
	NBR	20	3.683 ± 0.275 ^a^	3.117 ± 0.116 ^a^	3.700 ± 0.132 ^a^	3.650 ± 0.132 ^a^	3.000 ± 0.265 ^a^	0.0535
		10	6.883 ± 1.358 ^b^	6.050 ± 0.218 ^b^	7.567 ± 0.202 ^b^	7.717 ± 0.633 ^b^	6.367 ± 0.104 ^b^	0.0023 **
	UHMW	20	3.867 ± 0.076 ^a^	2.650 ± 0.000 ^a^	4.017 ± 0.076 ^a^	3.917 ± 0.104 ^a^	3.150 ± 0.050 ^a^	0.0000***
		10	8.217 ± 0.189 ^b^	6.083 ± 0.058 ^b^	7.867 ± 0.058 ^b^	8.033 ± 0.176 ^b^	6.783 ± 0.104 ^b^	0.0000 ***
	EPDM	20	3.450 ± 0.132 ^a^	2.917 ± 0.116 ^a^	3.550 ± 0.050 ^a^	3.767 ± 0.144 ^a^	3.267 ± 0.362 ^a^	0.0031 **
		10	6.983 ± 0.765 ^b^	5.833 ± 0.318 ^b^	7.550 ± 0.087 ^b^	7.750 ± 0.278 ^b^	6.650 ± 0.520 ^b^	0.0032 **
	NR	20	3.883 ± 0.340 ^a^	3.117 ± 0.231 ^a^	3.750 ± 0.218 ^a^	3.633 ± 0.104 ^a^	3.367 ± 0.116 ^a^	0.0001 ***
		10	8.300 ± 0.755 ^b^	5.783 ± 0.116 ^b^	7.600 ± 0.328 ^b^	7.283 ± 0.202 ^b^	6.650 ± 0.132 ^b^	0.0105 **

** *p* < 0.01 or *** *p* < 0.001 in rows indicates differences between the various materials for each diameter, in columns different superscript letters indicate significant differences between flow for the same gas and material. IIR: Butyl Rubber; EPDM: Ethylene Propylene Diene Monomer rubber; UHMW: Ultra-high-molecular-weight polyethylene and NBR: Nitrile Butadiene Rubber.

**Table 5:** Time required (min) to lower the oxygen partial pressure in a DN32 butyl rubber hose of different lengths with the different inert gases tested (*n* = 3).

Flow Rate (L/min)	Inert Gas	Lengths (m)			
		10 m	20 m	30 m	50 m
5	CO_2_	3.23 ± 0.08 ^b,c^	3.73 ± 0.12 ^b^	6.18 ± 0.37 ^c^	7.32 ± 0.55 ^a^
	N_2_	3.45 ± 0.13 ^c^	2.33 ± 0.08 ^a^	3.45 ± 0.00 ^a^	5.97 ± 0.03 ^a^
	Ar	3.33 ± 0.06 ^b,c^	2.47 ± 0.03 ^a^	3.78 ± 0.03 ^a^	6.12 ± 0.13 ^a^
	Ar/CO_2_	3.00 ± 0.15 ^b^	4.35 ± 0.09 ^b^	6.20 ± 0.25 ^c^	
	N_2/_CO_2_	2.15 ± 0.05 ^a^	2.45 ± 0.00 ^a^	5.30 ± 0.09 ^b^	
	*p* level	0.0015 **	0.0000 ***	0.0005 ***	0.1077
10	CO_2_	2.53 ± 0.03 ^b^	2.32 ± 0.12 ^b^	3.25 ± 0.13 ^c^	3.88 ± 0.08 ^a^
	N_2_	1.83 ± 0.15 ^ab^	1.62 ± 0.03 ^a^	2.22 ± 0.03 ^a^	3.80 ± 0.13 ^a^
	Ar	2.30 ± 0.10 ^c^	1.62 ± 0.03 ^a^	2.15 ± 0.00 ^a^	3.43 ± 0.10 ^a^
	Ar/CO_2_	2.07 ± 0.12 ^bc^	2.47 ± 0.03 ^b^	2.82 ± 0.03 ^b^	
	N_2/_CO_2_	1.62 ± 0.03 ^a^	1.45 ± 0.00 ^a^	2.62 ± 0.03 ^b^	
	*p* level	0.0069 **	0.0002 ***	0.0003 ***	0.1061
20	CO_2_	1.43 ± 0.08 ^c^	0.88 ± 0.03 ^ab^	1.32 ± 0.08 ^a^	2.03 ± 0.03 ^b^
	N_2_	1.00 ± 0.05 ^a^	1.02 ± 0.03 ^c^	1.45 ± 0.00 ^a^	1.72 ± 0.03 ^a^
	Ar	1.05 ± 0.00 ^ab^	0.97 ± 0.03 ^bc^	1.33 ± 0.06 ^a^	1.98 ± 0.03 ^b^
	Ar/CO_2_	1.20 ± 0.00 ^b^	1.12 ± 0.03 ^d^	1.67 ± 0.03 ^b^	
	N_2/_CO_2_	0.95 ± 0.00 ^a^	0.80 ± 0.00 ^a^	1.32 ± 0.03 ^a^	
	*p* level	0.0025 **	0.0024 **	0.0142 ***	0.010 *

* *p* < 0.05 or ** *p* < 0.01 or *** *p* < 0.001 in rows indicates differences between gases for length of the hose and flow rate, in columns different superscript letters indicate significant differences between gas for the same flow and length.

**Table 6:** Increase in the oxygen partial pressure (hPa) in the model wine during a racking in 1000 L and 1500 L tanks, with different inertization strategies and with different inert gases.

Gas	Blanketing Full Tank		Flushing Empty Tank		Both Tanks Inertized	
	1000 L	1500 L	1000 L	1500 L	1000 L	1500 L
CO_2_	11.67	12.32	10.33	9.32	11.08	13.84
N_2_	12.00	8.39	5.30	8.38	4.84	11.73
Ar	11.58	5.64	6.26	6.74	5.34	11.06
Ar/CO_2_	11.30	10.69	5.99	9.33	6.33	11.03
N_2/_CO_2_	12.22	6.00	6.06	8.87	7.39	12.69
No gas		14.92	12.61	15.13	11.80	15.07

**Table 7:** Increase in the oxygen partial pressure (hPa) in the model wine during filling through the bottom valve in a previously purged tank with different inertization gases and volumes.

Gas Volume	1500 L Tank (Ar)			1000 L Tank (CO_2_)		
	Initial pO_2_	Final pO_2_	ΔpO_2_	Initial pO_2_	Final pO_2_	ΔpO_2_
Half tank	1.431	5.345	3.914	0.736	5.206	4.470
Third tank	0.367	7.108	6.741	0.860	6.158	5.298
Quarter tank	1.322	8.528	7.206	1.462	8.419	6.957

**Table 8:** Oxygenation caused in the racking according to the different levels of inertization studied.

O_2_ Addition to Wine	No Inert Gases	Hoses Purged (N_2_)	Tank Flushed (CO_2_)	Full Flushing & Blanketing
hPa	12.80	8.40	4.30	0.25
% air sat.	6.70	4.39	2.23	0.13
% O_2_	1.39	0.92	0.47	0.03
mg/L	0.56	0.38	0.18	0.01

**Table 9:** Comparison of the OTR levels of the different seal materials in relation to the FKM/FPM in the two types of connectors and for each of the diameters studied.

Material	DIN 11851			Tri-Clamp		
	DN32	DN50	DN80	DN32	DN50	DN80
PTFE	3294	3227	1475	150	718	2011
NBR	4	2	0.2	2	1	1
FKM/FPM	1	1	1	1	1	1
Q/VMQ	194	1491	4	110	84	5
EPDM	5	5	1	4	3	1

PTFE: Polytetrafluoroethylene; NBR: Nitrile Butadiene Rubber; FKM/FPM: Fluorocarbon; Q/VMQ: silicone rubber and EPDM: Ethylene Propylene Diene Monomer rubber.

**Table 10:** Comparison of the inert gas volume for a complete purge index as well as the cost for the inert gases tested in the different hoses studied.

		**Gas**				
	**Gas Properties**	**CO_2_**	**Ar**	**N_2_/CO_2_**	**N_2_**	**Ar/CO_2_**
	Density (g/L)	1.840	1.669	1.300	1.169	1.410
	Specific gravity *	1.502	1.362	1.061	0.955	1.151
	Gas cost €/L	0.0040	0.0116	0.0109	0.0065	0.0130
**Hose material**	**Inertization data**	**CO_2_**	**Ar**	**N_2_/CO_2_**	**N_2_**	**Ar/CO_2_**
IIR	I_va/vg_	0.8857	1	1.0128	1.0225	1.0678
	Purge cost (10^−3^ €/L)	3.54	11.59	11.00	6.67	13.87
	R^2^	0.9314	0.8984	0.9852	0.9275	0.9899
NBR	I_va/vg_	0.8872	1.0501	1.0062	1.0534	1.1099
	Purge cost (10^−3^ €/L)	3.54	12.17	10.92	6.87	14.42
	R^2^	0.9833	0.9967	0.9889	0.9957	0.993
EPDM	I_va/vg_	0.9583	1.0496	1.1751	0.9433	1.0839
	Purge cost (10^−3^ €/L)	3.83	12.16	12.76	6.16	14.08
	R^2^	0.9912	0.9949	0.9969	0.9976	0.9975
UHMW	I_va/vg_	1.1035	1.1615	1.1107	0.9702	1.1594
	Purge cost (10^−3^ €/L)	4.41	13.46	12.06	6.33	15.06
	R^2^	0.9952	0.9972	0.9947	0.9857	0.9978

* Based on the air density = 1.225 g/L; I_va/vg_ relationship between the volume of purged air and the volume of inert gas used to displace the air; R^2^: coefficient of determination. IIR: Butyl Rubber; NBR: Nitrile Butadiene Rubber; EPDM: Ethylene Propylene Diene Monomer rubber and UHMW: Ultra-high-molecular-weight polyethylene.

## Data Availability

The data presented in this study are available on request from the corresponding author. The data are not publicly available due to the fact that they belong to the different companies collaborating in the cession of the materials.

## Supplementary Materials

The following are available online at https://www.mdpi.com/2304-8158/10/2/386/s1, Figure S1: Time needed to evacuate the air from the interior of DN32 hoses of 10 m with different gases at three different flows 2.5, 5 and 10 L/min (a) EPDM; (b) NBR and (c) UPE. Figure S2: Time needed to evacuate the air inside 30 m DN50 hoses with different gases at two different flow rates 5 and 10 L/min (a) EPDM; (b) NBR; (c) UPE and (d) NR
