# Supplementary material for: Characterization and Control of Hidden Micro-Oxygenation in the Winery: Wine Racking
Source: Foods. 2021 Feb 10;10(2):386. doi: 10.3390/foods10020386 (PMC7916471; doi:10.3390/foods10020386)
Supplement: Supplementary file 1 [file foods-10-00386-s001.pdf]

Supplementary material

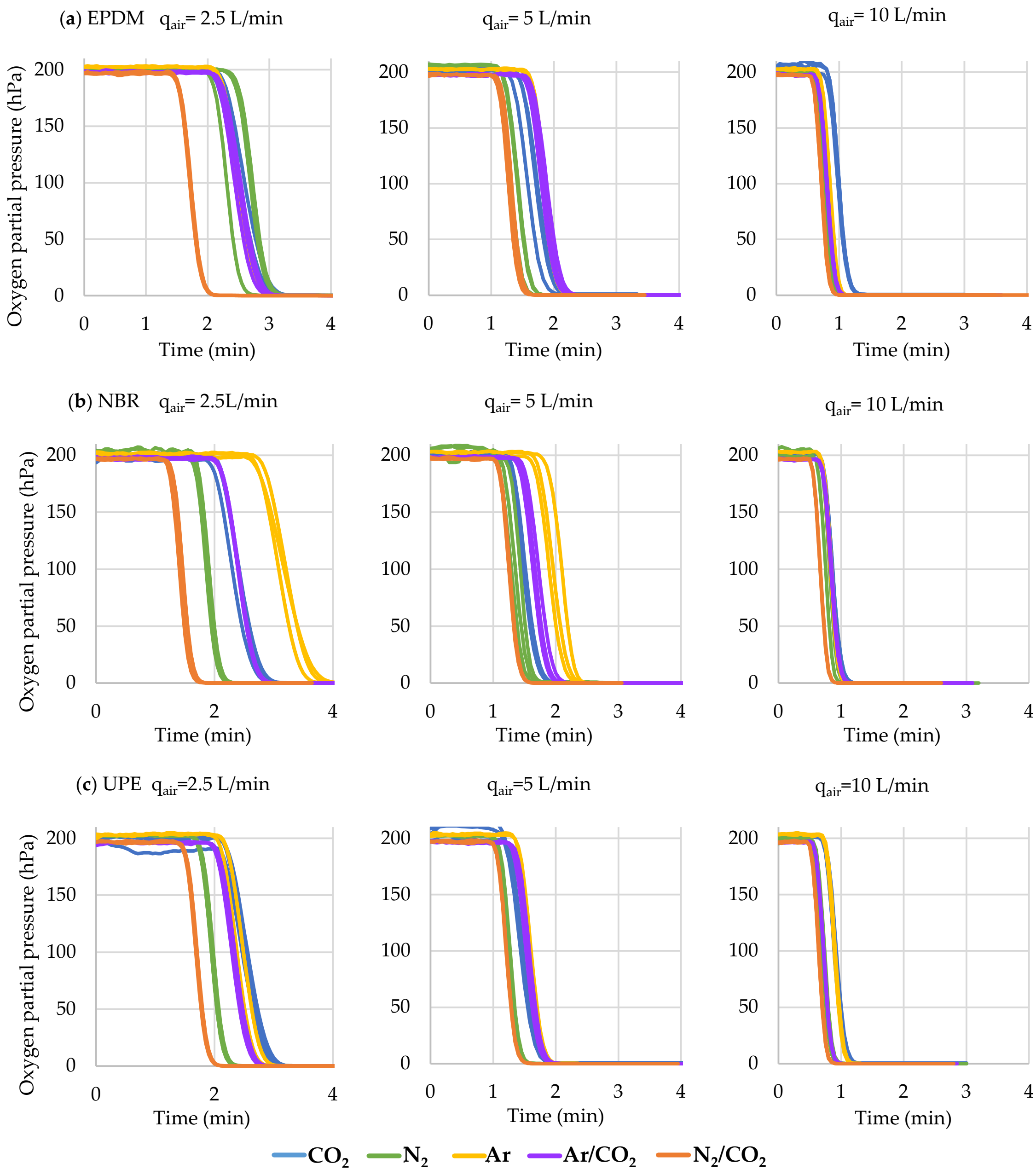

Figure S1. Time needed to evacuate the air from the interior of DN32 hoses of 10 m with different gases at three different flows 2.5, 5 and 10 L/min (**a**) EPDM; (**b**) NBR and (**c**) UPE.

Supplementary material

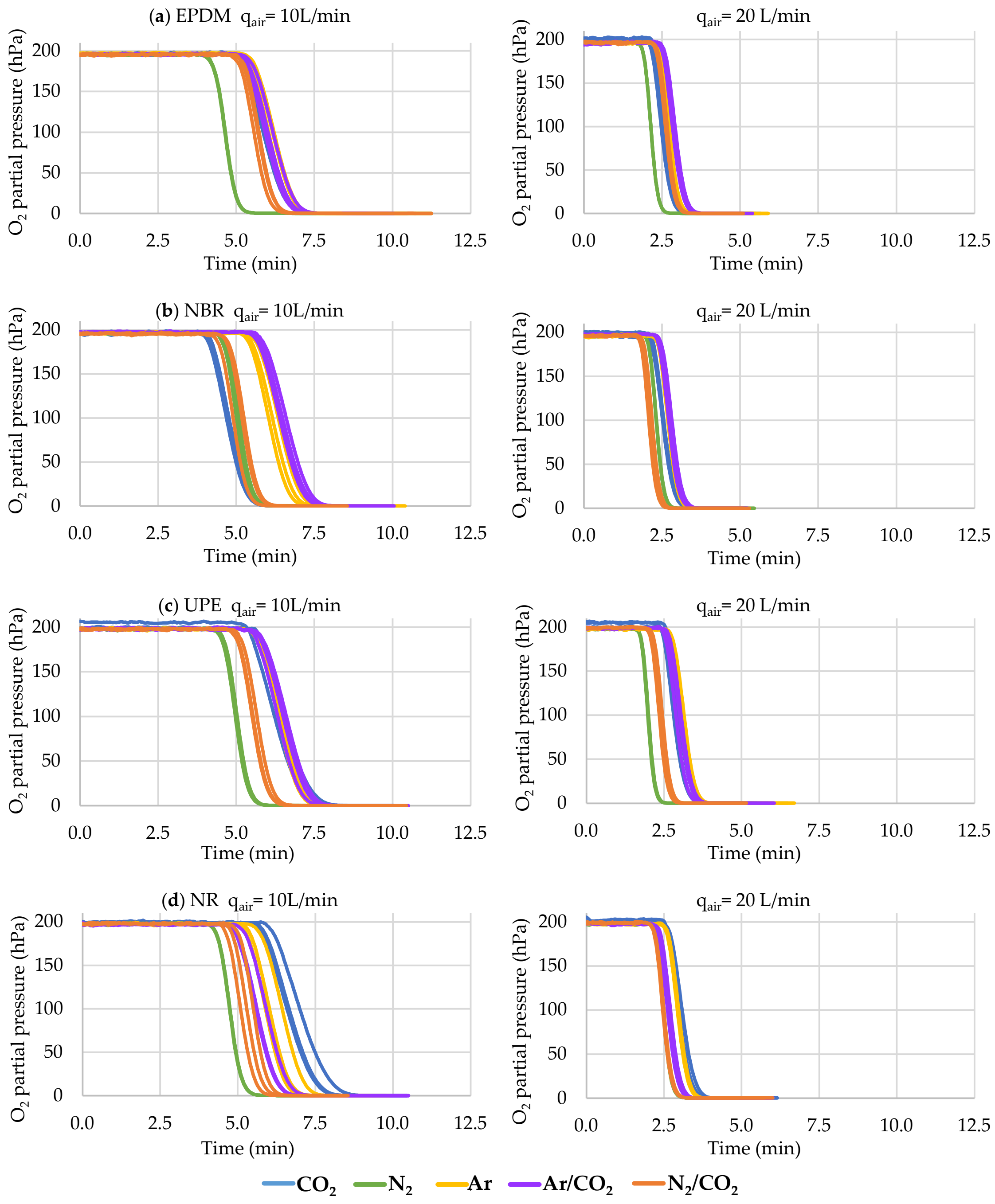

Figure S2. Time needed to evacuate the air inside 30 m DN50 hoses with different gases at two different flow rates 5 and 10 L/min (**a**) EPDM; (**b**) NBR; (**c**) UPE and (**d**) NR.
